# Supplementary material for: Patterns of Intron Gain and Loss in Fungi
Source: PLoS Biol. 2004 Nov 30;2(12):e422. doi: 10.1371/journal.pbio.0020422 (PMC532390; doi:10.1371/journal.pbio.0020422)
Supplement: Table S1 — Also available at http://genes.mit.edu/NielsenEtAl/. (4.3 MB ZIP). [file pbio.0020422.st001.zip › NielsenEtAl/html/132.html]

AN1177.1.NCU04404.1.MG06860.1.FG02780.1


```
 CLUSTAL W (1.82) Multiple Sequence Alignments - Introns Inserted


Sequence 1: NCU04404.1	957 aa
Sequence 2: FG02780.1	957 aa
Sequence 3: MG06860.1	958 aa
Sequence 4: AN1177.1	950 aa
Alignment Length: 958 aa
Number Identitical Residues: 722 aa
Alignment Score (without introns) 29255


MG06860.1 	MSGFLENAYSLVHQDNAADVPSLNDLRTQLEKGTDETKVDTMKRILTIMLNGDPMPSLLM
NCU04404.1	MS-FLENAYSLVKDNAVDGTPTVQELKTQLEKGTDETKIETMKRILTIMLNGDPMPQLLM
FG02780.1 	MSGFLENAYSLVHQDNAADVPTVSDLRMQLEKGTDESKVETMKRILTIMLNGDPMPSLLM
AN1177.1  	MASFLENAYSLVHMDNAADQPSQQELKLQLEKGTDETKLDTMRRIITIMLNGDPMPQLLM
          	*:.*********: : . . *: .:*: ********:*::**:**:**********.***

MG06860.1 	HIIRFVMPSKSKHLKKLLYFYYEICPKLDAQGKLKQEMILVC2NGIRNDLQHPNEYIRGN
NCU04404.1	HIIRFVMPSKSKPLKKLLYFYYEICPKLDAQGKLKQEFILVC2NGIRNDLQHPNEYIRGN
FG02780.1 	HIIRFVMPSKYKPLKKLLYFYYEICPKLDSTGKLKQEMILVC2NGIRNDLQHPNEYIRGN
AN1177.1  	HIIRFVMPSKSKPLKKLLYFYYEICPKHDSNGKLKQEMILVC2NGIRNDLQHPNEYVRGN
          	********** * ************** *: ******:**** *************:***

MG06860.1 	TLRFLCKLREAELIEPLLSSARSCLEHRHAYVRKNAVFAVASIFQHSPSLIPDAADLIAT
NCU04404.1	TLRFLCKLREAELLEPLLSSARGCLEHRHAYVRKNAVFAVASIYQHSPSLIPDAADLIST
FG02780.1 	TLRFLCKLREAELIEPLLSSARSCLEHRHAYVRKNAVFAISSIHTHSPSLIPDASELIST
AN1177.1  	TLRFLCKLREPELIEPLLSSARSCLDHRHAYVRKSAVWAISSIFQHSESLIPDAPELIQT
          	**********.**:********.**:********.**:*::**. ** ******.:** *

MG06860.1 	FLESESDPTCKRNAFAALASISHDHALIYLSSVFDGIPNAEELLQLVELEFIRKDAVQNS
NCU04404.1	FLEGESDPTCKRNGFAALASIDHDKALLYLSTVFDGIPNADELLQLAELEFIRKDAVVNT
FG02780.1 	FLEGESDGVCRRNGFAALASIDHDAALLYLSSVFEGIPNAEELLQLVELEFIRKDAVQNS
AN1177.1  	FLETESDGTCKRNAFAALMSISHQKALEYLASTFDSIPNTDELLQLAELEFIRKDAVQNS
          	*** *** .*:**.**** **.*: ** **::.*:.***::*****.********** *:

MG06860.1 	QNK0ARYLRLIFDLLEAGASTVVYEAASSLTALTSNPVAVKAAAAKFIELSIKEADNNVK
NCU04404.1	QNK~ARYLRLIFDLLEASASTVIYEAASSLTALTNNPVAVKAAASKFIELAIKEADNNVK
FG02780.1 	QNK0ARYLRLIFDLLEAGASTVVYEAASSLTALTNNPVAVKAAAAKFIELSIKEADNNVK
AN1177.1  	QNK0ARYLRLIFDLLDASTSTVVYEAATSLTALTSNPVAVKAAAGKLIELSIKEADNNVK
          	*** ***********:*.:***:****:******.*********.*:***:*********

MG06860.1 	LIVLDRVDQLRKKNEGVLDDLVMEILRVLSSPDIDVRRKALGIALDMVSSKNVEEVVLLL
NCU04404.1	LIVLDRVDQLRQKNPGVLDDLTMEVLRVLSSTDIDVRRKGLEIALEMVSSKNVEEVVLLL
FG02780.1 	LIVLDRVDQLRKKNEGVLDDLTMEILRVLSSTDIDVRRKALGLALEMVSSKNVEEVVLLL
AN1177.1  	LIVLDRVDQLRIRNEGVLDDLTMEILRVLSSPDIDVRRKALGIALEMVSSKNVEEIVMLL
          	*********** :* ******.**:******.*******.* :**:*********:*:**

MG06860.1 	KKELSKTVDQEYEK~NSEYRQLLIHSIHQCAIKFSEVAASVVDLLMDFIADFNNTSSVDV
NCU04404.1	KKELSKTVDQEYEK~NNEYRQLLIHSIHQCAIKFSEVAASVVGLLMDFIADFNNNSAVDV
FG02780.1 	KKELSKTVDQEYEK~NTEYRSLLIHSIHQCAIKFSEVAASVVELLMDFIADFNNVSAVDV
AN1177.1  	KKELSKTVDEQYEK0NSEYRQLLIQSIHNCAIKFSEIAASVVDLLMDFIADFNNNSAVDV
          	*********::*** *.***.***:***:*******:***** *********** *:***

MG06860.1 	INFVKEVVEKFPTLRPSIVTRLVETLAEVRAGKVYRGILWIIGEYSLEEKDIRDAWRRIR
NCU04404.1	INFVKEVVEKFPQLRPAIIARLVDTLSEVRAGKIYRGILWIIGEYSLEEKDIRDAWKRIR
FG02780.1 	INFVKEVVEKFPNLRTTIIERLVSTLGEVRAGKVYRGIMWIIGEYSLEEKDIREAWKRIR
AN1177.1  	ISFVKEVVEKFPKLRPSIVNRLVSTLSEVRAGKVYRGVLWVVGEYSLEESDIREAWKKIR
          	*.********** **.:*: ***.**.******:***::*::*******.***:**::**

MG06860.1 	ASLGEIPILASEQRLLDTADEEDSKADDQVNGGSKAAPTGSRKVLADGTYATETALTSQS
NCU04404.1	ASLGEIPILASEQRLLDNVDGDAEEKEKEQVNGSRAAPTGSRKVLADGTYATETALTSQS
FG02780.1 	ASLGEMPILASEQRLLDSHEGDEKTEDHTNGTSKPAAPSGSRKVLADGTYATETALTSQS
AN1177.1  	ASLGEIPILASEQRLLEEVPEDTLPKEQINGHG-KSAP----KVLADGTYATESALTSQS
          	*****:**********:    :    :.    .  :**    ***********:******

MG06860.1 	SKAAKLEAVKAAQKPPLRQLILDGDYYLATVLSSTLTKLVMRHSEISSDHARTNALRAEA
NCU04404.1	TAAARLEAVKASSKPPLRQLILDGDYYLATVLASTLTKLVMRHAEISSEEARTNALRAEA
FG02780.1 	SAAARLEAVKTAQKPPLRQLILDGDYYLATVLSSTLVKLVMRHHEISSDKARTNALRAEA
AN1177.1  	AAAARLQAVKAAQKPPLRQLILDGDYYLATVLSSTLTKLVMRHSEVSEDSARTNALRAEA
          	: **:*:***::.*******************:***.****** *:*.: **********

MG06860.1 	MLIMISIIRVGQSQFVKAPIDEDSVDRIMSCVRSLAEFTERKELETVYLEDTKKAFRAMV
NCU04404.1	MLIMISVIRVGQSQFVKAPIDEDSVDRIMSCVRSLAEFTAHKELETVYLEDTRKAFRAMV
FG02780.1 	MLIMISIIRVGQSQFVKAPIDEDSVDRIMSCVRSLAEFEEKKELETVWLDDTRKAFRAMV
AN1177.1  	MLIMISIIRVGQSPFVAAPIDEDSVDRIMTCVRSLAEFSERKELETTFLEDTRKAFRAMV
          	******:****** ** ************:********  :*****.:*:**:*******

MG06860.1 	AVEEKKRAAKVAVEKAKTAVQVDDVVAIRQLSKKNAGDGADMAEVDLERATGGDSSAAED
NCU04404.1	QVEEKKRAAKEAHQKAKTATQVDDVYQIRQLSKKNATDGADAFDADLERATGGDNSSAED
FG02780.1 	QVEEKKRAAKEAFEKAKTAVQVDDVVSIRQLAKKNTTDGLDEIEVDLERATGGE-GAAED
AN1177.1  	QVEDKKRAAKEAVEKAKTAVQIDDAIPIRQFAKKSALEGAEEIELDLAKATGGD-SAVET
          	 **:****** * :*****.*:**.  ***::**.: :* :  : ** :****: .:.* 

MG06860.1 	LSSKLNRVVQLTGFSDPVYAEAYVKVHQFDIILDVLLVNQTTETLQNLSVEFATLGDLKV
NCU04404.1	LSGKLSRVVQLTGFSDPVYAEAYVKVHQFDIVLDVLLVNQTTETLQNLTVEFATLGDLKV
FG02780.1 	LSSKLSRVVQLTGFSDPVYAEAYVKVHQFDIVLDVLLVNQTTETLQNLSVEFATLGDLKV
AN1177.1  	VSSKLSRVVQLTGFSDPVYAEAYVTVHQFDIVLDVLLVNQTLETLQNLSVEFATLGDLKV
          	:*.**.******************.******:********* ******:***********

MG06860.1 	VERPTTQNLGPHDFHNVQCTIKVSSTDTGVIFGNVVYDGAHSTDTHVVILSDVHVDIMDY
NCU04404.1	VEKPTSQNLGPHDFHNVQCTIKVSSTDTGVIFGNVVYEGAHSTDTHVVILNDLHVDIMDY
FG02780.1 	VERPTTQNLGPHDFHNVQCTIKVSSTDTGVIFGNVVYDGAHSTDTNVVILNDLHVDIMDY
AN1177.1  	VERPTTHNLGPRDFLNVQATVKVSSTDTGVIFGNIVYDGASSTETHVVILNDIHADIMDY
          	**:**::****:** ***.*:*************:**:** **:*:****.*:*.*****

MG06860.1 	IQPATCTETQFRTMWTEFEWENKVNINSKIKTLRGFLDELMKATNMNCLTPEAGLKGDCQ
NCU04404.1	IQPATCTETQFRTMWTEFEWENKVNINSKAKSLREFLDLLMASTNMNCLTPEASLKGDCQ
FG02780.1 	IQPATCTETQFRTMWTEFEWENKVNINSKAKTLRDFLDQLMACTNMNCLTPEASLKGDCQ
AN1177.1  	IQPAHCTETQFRTMWTEFEWENKVNINSKAKSLRDFLKQLMESTNMACLTPEASLTGDCR
          	**** ************************ *:** **. ** .*** ******.*.***:

MG06860.1 	FLSANLYARSVFG1EDALANLSIEKEGEDGPITGFVRIRSRSQGLALSLGSLKGLTKIGT
NCU04404.1	FLSANLYARSVFG1EDALANLSIEKEGEDGPITGFLRIRSRSQGLALSLGSLKALNKIGS
FG02780.1 	FLSANLYARSVFG1EDALANLSIEKEGEDGPITGFVRIRSRSQGLALSLGSLKGLNKIGS
AN1177.1  	FLSANLYARSVFG1EDALANLSIEKEGEDGPVTGFVRIRSRSQGLALSLGSLKGLKASAA
          	************* *****************:***:*****************.*.  .:

MG06860.1 	SS
NCU04404.1	AA
FG02780.1 	TS
AN1177.1  	--
          	
```
